# Life Sciences New Talent collection

**DOI:** 10.1098/rsos.211981

**Published:** 2022-01-19

**Authors:** Catrin Pritchard, Malcolm F. White, Steve Brown

**Affiliations:** ^1^ Cancer Studies, University of Leicester College of Medicine Biological Sciences and Psychology, Leicester LE1 9HN, UK; ^2^ School of Biology, University of St Andrews, St Andrews, UK; ^3^ Mammalian Genetics Unit, MRC Harwell Institute, Oxfordshire, UK

We introduce the Life Sciences New Talent collection, comprising invited papers which showcase emerging fields of cross-disciplinary life sciences research and some of the exciting work funded by the Royal Society. The collection is part of a series of commissioned New Talent collections originally started with the Chemistry New Talent collection [1] which had its genesis in the unique collaboration between the Royal Society of Chemistry and *Royal Society Open Science* [2].

We, the Editors of the Biochemistry, Molecular & Cellular Biology, and Genetics & Genomics sections of *Royal Society Open Science*, commissioned this collection with the aim of expanding the breadth of these New Talent collections to showcase early career investigators in the Life Sciences.

The papers published in this collection were invited from recent awardees of a Royal Society University Research Fellowship (URF) [3], Sir Henry Dale Fellowship [4] or Dorothy Hodgkin Fellowship [5]. These awards are granted via a competitive grant application process to allow outstanding early stage scientists to build their own independent research career addressing an important biological or biomedical question.

We have enjoyed reading and preparing these manuscripts, which highlight the extraordinary breadth of new talent in the UK Life Sciences research community. Yulia Yuzenkova, a Royal Society URF at Newcastle University, reviews the fast-developing field of non-canonical RNA capping, highlighting the prevalence of this RNA modification across the domains of life [6]. Lu-Ning Liu, a Royal Society URF at the University of Liverpool, explores the complexity of photosynthetic complexes in cyanobacteria, providing new insights on the interactions between these complexes in the thylakoid membrane [7]. Thomas Richards, a Royal Society URF at the University of Oxford, presents research from his group focused on characterization of the RNA-interference pathways of *Paramecium bursaria,* opening the door to the investigation of its nascent endosymbiotic relationship with a green algae partner [8]. Michael Morrissey, a Royal Society URF at the University of St Andrews, and colleagues report on their research to apply new deep-learning techniques for re-identification of individuals in wild populations, (in their case the Arctic charr) an area that is fundamental to ecological and evolutionary studies [9]. Benjamin Hall, a Royal Society URF at University College London, and colleagues identify an approximate Bayesian computation-based approach as the most accurate for estimating uncertainties in lineage tracing datasets, thus informing experimental design in transgenic systems [10].

Helge Dorfmueller, a Sir Henry Dale Fellow at Dundee University, contributes a review of recent developments in the understanding of Group A Streptococcus pathogenesis and the implications for vaccine development [11]. Kok-Lung Chan, a Sir Henry Dale Fellow at the University of Sussex, reviews the causes and consequences of incomplete genomic DNA replication and the role of replication stress on genomic instability [12]. Thomas Walker, a Sir Henry Dale Fellow at the London School of Hygiene and Tropical Medicine, presents research from his group on novel *Wolbachia* bacterial strains in populations of the mosquito *Anopheles gambiae* in Guinea—findings that identify further candidate strains that might be used in the future for malaria biocontrol strategies [13]. Christos Gkogkas, a Sir Henry Dale Fellow at the University of Edinburgh, contributes a timely review of recent developments in translational controls in brain development in neurons and endothelial cells, and implications for the treatment of neurodevelopmental disorders [14].

At the time of writing, we also hope to include the following contributions to our New Talent collection: Andrew Hitchcock, a Royal Society URF at the University of Sheffield, and colleagues review the state of our understanding of the catalytic function, substrate recognition and regulation of two key enzymes which are critical to the final stages of (bacterio)chlorophyll biosynthesis. Benjamin Steventon, a Sir Henry Dale Fellow at the University of Cambridge, reviews the current state-of-the art in the study of pattern formation in static and motile cellular environments, looking ahead to challenges and the approaches that may surmount them. Maria Christophorou, a Sir Henry Dale Fellow at the Babraham Institute, reviews citrullination and its consequences for cell physiology. Joe Grove, a Sir Henry Dale Fellow at the Centre for Virus Research, Glasgow, surveys processes of viral entry to cells and mechanisms of inhibition by antibodies.

We hope that readers of the journal will explore this collection and find something of interest. These papers are all curated at https://royalsocietypublishing.org/topic/special-collections/life-sciences-new-talent [15].
